# Supplementary figures and images for: Examination of the role of mutualism in immune evasion
Source: Front Oncol. 2024 May 8;14:1406744. doi: 10.3389/fonc.2024.1406744 (PMC11109368; doi:10.3389/fonc.2024.1406744)

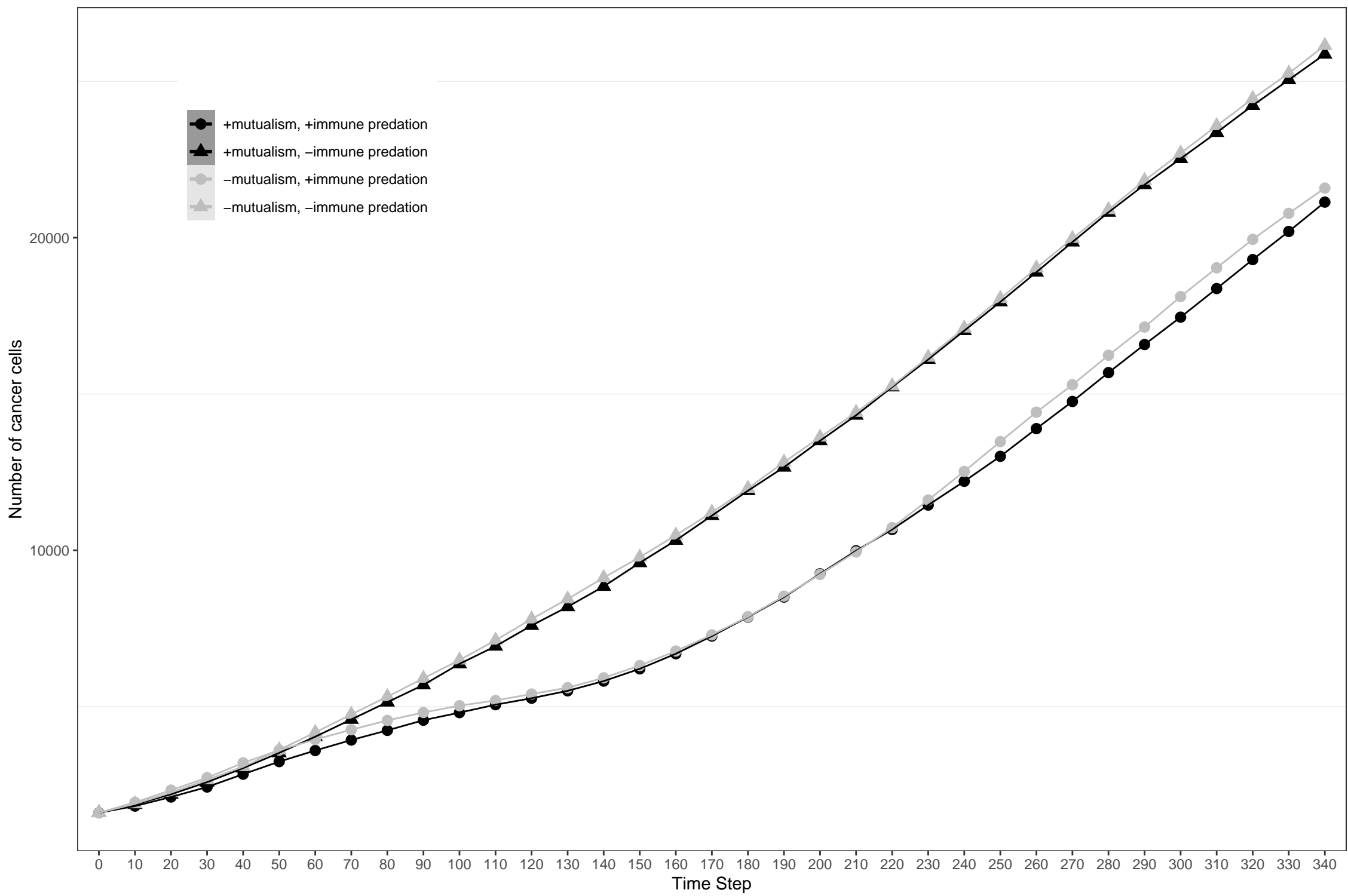

Supplement: Supplementary Figure 1 — Number of cancer cells depending on different conditions, the division rate in the simulation is 0.09. [file Image_1.pdf]

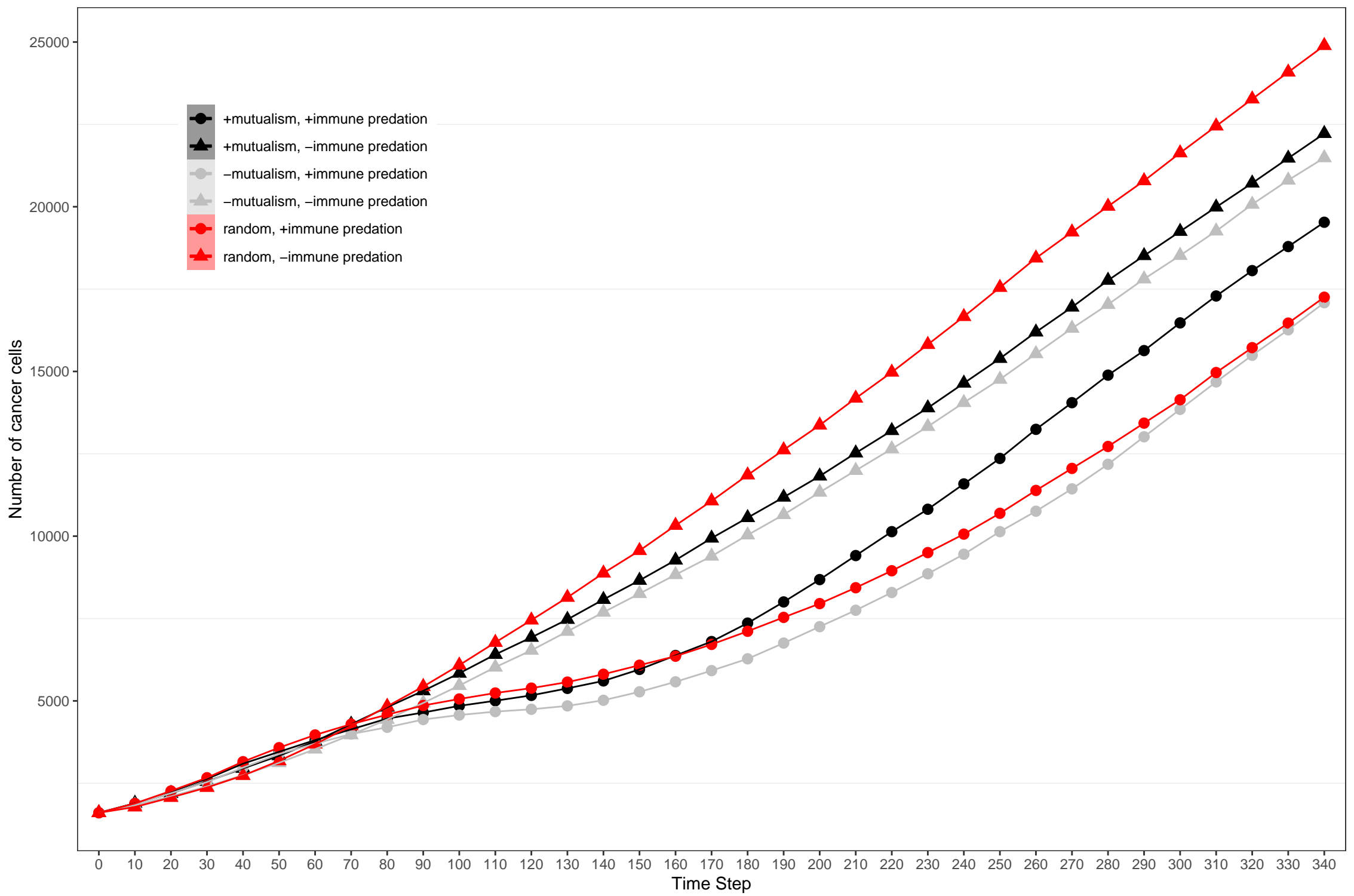

Supplement: Supplementary Figure 2 — Number of cancer cells for random mutualism, the division rate in the simulation is 0.08. [file Image_2.pdf]

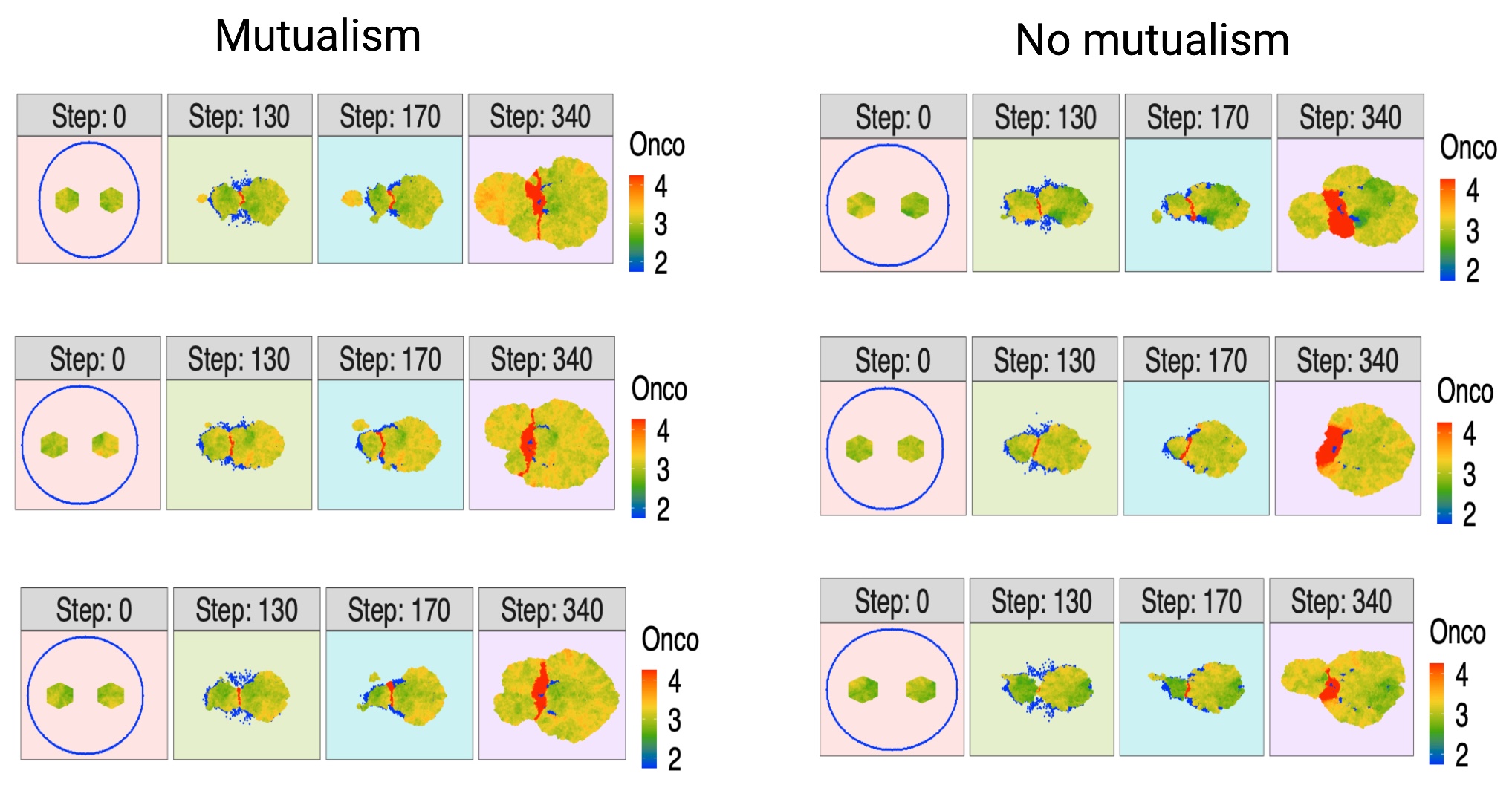

Supplement: Supplementary Figure 3 — Qualitative comparison of mutualism and no mutualism. On the left simulations supporting our hypothesis, on the right simulations which do not support our hypothesis. [file Image_3.jpg]
